# Detection and Characterization of Protein Interactions *In Vivo* by a Simple Live-Cell Imaging Method

**DOI:** 10.1371/journal.pone.0062195

**Published:** 2013-05-01

**Authors:** Oriol Gallego, Tanja Specht, Thorsten Brach, Arun Kumar, Anne-Claude Gavin, Marko Kaksonen

**Affiliations:** 1 Institute for Research in Biomedicine-IRB, Barcelona, Spain; 2 European Molecular Biology Laboratory, Heidelberg, Germany; Thomas Jefferson University, United States of America

## Abstract

Over the last decades there has been an explosion of new methodologies to study protein complexes. However, most of the approaches currently used are based on *in vitro* assays (e.g. nuclear magnetic resonance, X-ray, electron microscopy, isothermal titration calorimetry etc). The accurate measurement of parameters that define protein complexes in a physiological context has been largely limited due to technical constrains. Here, we present PICT (Protein interactions from Imaging of Complexes after Translocation), a new method that provides a simple fluorescence microscopy readout for the study of protein complexes in living cells. We take advantage of the inducible dimerization of FK506-binding protein (FKBP) and FKBP-rapamycin binding (FRB) domain to translocate protein assemblies to membrane associated anchoring platforms in yeast. In this assay, GFP-tagged prey proteins interacting with the FRB-tagged bait will co-translocate to the FKBP-tagged anchor sites upon addition of rapamycin. The interactions are thus encoded into localization changes and can be detected by fluorescence live-cell imaging under different physiological conditions or upon perturbations. PICT can be automated for high-throughput studies and can be used to quantify dissociation rates of protein complexes *in vivo*. In this work we have used PICT to analyze protein-protein interactions from three biological pathways in the yeast *Saccharomyces cerevisiae*: Mitogen-activated protein kinase cascade (Ste5-Ste11-Ste50), exocytosis (exocyst complex) and endocytosis (Ede1-Syp1).

## Introduction

Since specific recognition between proteins governs cellular function, the systematic study of protein-protein interactions (PPIs) has become a central endeavor in the fields of structural and cell biology [1]. However, although the crowded cellular environment is expected to play a major role in protein function, PPIs are usually studied using *in vitro* methods with isolated proteins. The *in vitro* approaches are also laborious and often not feasible with complexes that are difficult to purify. In vivo methods, on the other hand, allow one to study PPIs in the cellular context, but are usually technically challenging (e.g. Förster resonance energy transfer (FRET) [2] and fluorescence cross-correlation spectroscopy [3]) or are non-quantitative and prone to errors due to indirect readouts from reconstitution of the reporter tags (e.g. yeast two-hybrid [4] and other protein-fragment complementation assays [5]–[8]). All these limitations have traditionally restricted the accurate characterization of physiological PPIs. Here we describe a novel method, PICT (Protein interactions from Imaging of Complexes after Translocation), to detect and quantitatively characterize PPIs, both stable and transient, under different physiological states or upon genetic, chemical or environmental perturbations. PICT is based on the heterodimerization of FRB and FKBP proteins induced by the drug rapamycin [9]. A similar concept is used in the “anchor-away” method to conditionally inactivate proteins [10]. In the PICT assay a subunit of the complex of interest tagged to FRB (bait) is translocated to small static sites marked by a membrane-associated protein tagged to FKBP (anchor). When the heterodimerization of the FRB and FKBP domains is induced by rapamycin, GFP-tagged proteins (preys) interacting directly or indirectly with the bait co-translocate to the anchor site, and the change in their localization can be easily visualized. PICT can be combined with other light microscopy methods, such as fluorescence recovery after photobleaching (FRAP), to complement the characterization of PPIs in living cells. Importantly, PICT provides a very simple readout and can, therefore, be easily automated for high-throughput studies. To illustrate the potential of PICT, we have analyzed three different protein complexes expressed from their genomic loci in yeast: the Ste5-Ste11-Ste50 complex, the exocyst complex and the Ede1-Syp1 complex.

## Results and Discussion

### Using induced translocation to detect protein interactions *in vivo*

**Figure 1:**
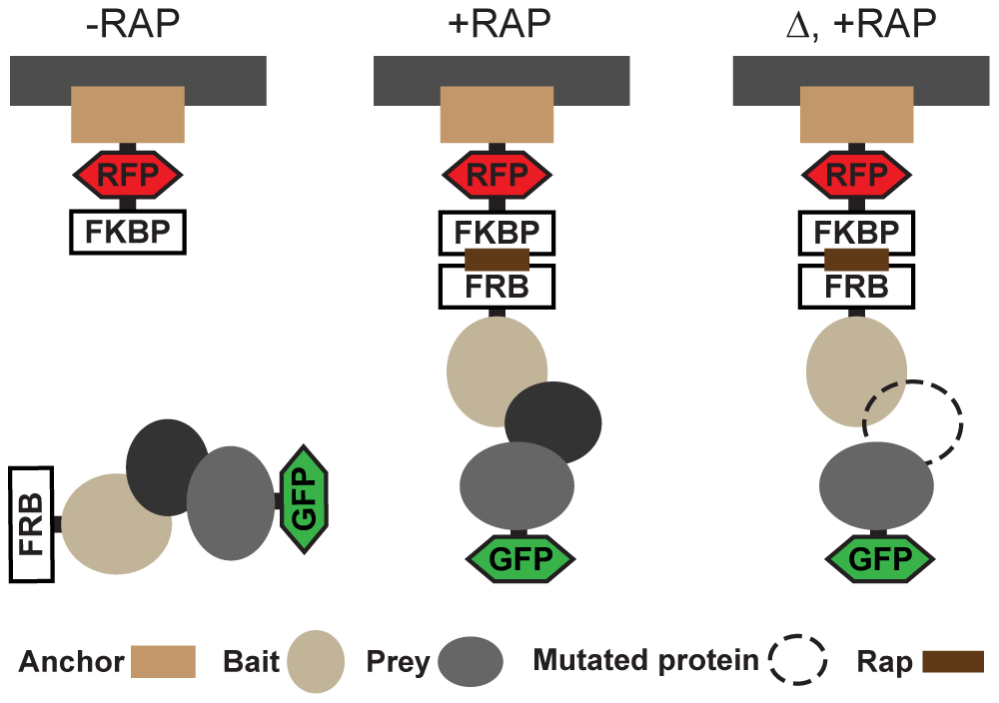
Representation of PICT approach. Schematic representation of the assay. “−RAP” cells were treated with the vehicle; “+RAP” cells were treated with rapamycin; “Δ, +RAP” denotes that a gene has been deleted and the cells have been treated with rapamycin.

PICT is based on chemically induced translocation of the target complex to a static cellular anchor site. The method depends on three components expressed in genetically engineered cells: anchor, bait and prey (Figure 1). The anchor is a protein that is stably bound to a distinct cellular location and is fused to red fluorescent protein (RFP) and the FKBP domain. The bait protein, a component of the studied complex, is fused to the FRB domain. Since FRB and FKBP form tight heterodimers in the presence of the drug rapamycin [9], anchor and bait heterodimerization is induced in cells exposed to rapamycin, which leads to relocalization of the bait protein to the anchor sites (Figure 1). GFP-tagged prey proteins interacting directly or indirectly with the bait will be co-recruited to the anchor sites as well. Consequently, PPIs between the bait and the prey are encoded into localization changes that can be directly visualized by fluorescence microscopy. Colocalization with the RFP-tagged anchor can be used to confirm and quantify the co-recruitment of the prey. Perturbations such as mutations in the components of the complex can be easily added to test the hierarchy of the PPIs (Figure 1).

PICT is applicable to any prey and bait proteins that can be tagged without interfering with their complex formation. The method requires that both bait and prey can be translocated to the anchor site, i.e., that the proteins are not tightly bound to some cellular structure or confined to a different cellular compartment. PICT may miss interactions that are dependent on a specific location inside the cell. Also, interactions of prey proteins that are expressed at much higher levels than the bait may be challenging to detect. However, as the method is based on colocalization it does not impose any steric requirements on the tags, unlike protein fragment complementation assays or FRET.

In this study we used Pil1 as an anchor protein (Pil1-RFP-FKBP) in the yeast *Saccharomyces cerevisiae*. Pil1 is a BAR domain protein that oligomerizes and binds tightly to distinct sites at the plasma membrane [11]. Anchor oligomerization and limited localization in small static sites increases the sensitivity of PICT for the detection of transient or rare binding events.

### Detecting PPIs and Mapping their hierarchy in a complex

**Figure 2:**
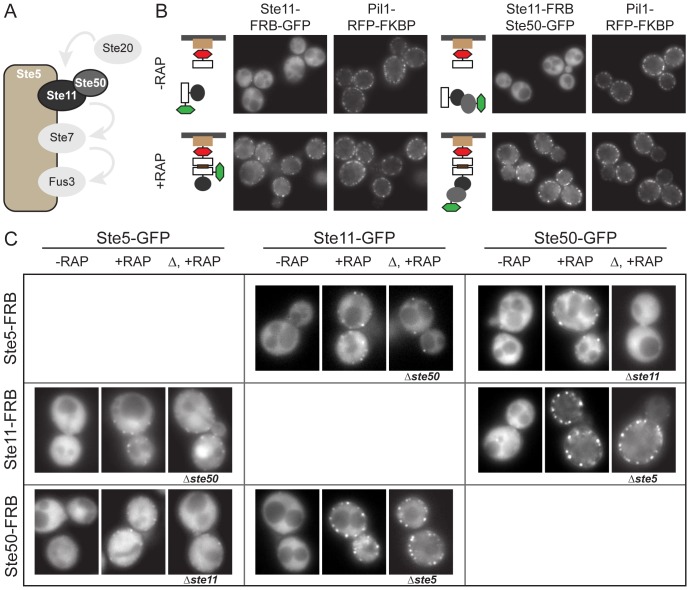
Analysis of the Ste5-Ste11-Ste50 MAPK cascade subcomplex with PICT. (A) Schematic representation of the Ste5-Ste11-Ste50 assembly. (B) Recruitment of the Ste5-Ste11-Ste50 complex to Pil1-RFP-FKBP anchoring platforms. Ste11-FRB was used as bait. Bait recruitment upon addition of rapamycin was proved in a strain in which Ste11 was tagged with FRB and GFP (left panel). Co-recruitment of Ste50-GFP prey is shown in the right panel. (D) Matrix with representative cells in the GFP channel for each of the six combinations resulting from all components of the studied complex used as bait (FRB-tagged) and prey (GFP-tagged) in PICT assays. (B) and (C) are color-coded as in Figure 1. “RAP” cells were treated with the vehicle, “+RAP” cells were treated with rapamycin. In (C) “▵, +RAP” denotes that the indicated gene has been deleted and the cells have been treated with rapamycin.

We first challenged PICT to characterize the Ste5-Ste11-Ste50 complex, a subcomplex of the Mitogen-activated protein kinase (MAPK) cascade, in living cells (Figure 2A). In normally growing haploid yeast cells Ste5, Ste11 and Ste50 have a predominantly cytosolic localization (Figures 2B and C). To first demonstrate the rapamycin-induced recruitment of the bait to the anchor we fused both FRB and GFP to Ste11. Upon rapamycin addition Ste11-FRB-GFP was translocated from its cytosolic localization to the anchor sites (Figure 2B). Similarly, when Ste11-FRB was used as bait, the prey Ste50-GFP was co-translocated to the anchor site indicating that Ste11 and Ste50 interact in vivo (Figure 2B; see also Video S1).

We then combined genetic perturbations with PICT to study the hierarchy of the PPIs in the Ste5-Ste11-Ste50 complex. We applied the PICT assay on the Ste5-Ste11-Ste50 complex systematically using all possible combinations of baits and preys. Additionally, we performed PICT in strains lacking one of the subunits (Figure 2C). Thus, the recruitment of those subunits that assemble through interactions mediated by the deleted proteins should be impaired (Figure 1). Before perturbation, all preys were successfully co-recruited, independently of which bait was used, demonstrating that the Ste5-Ste11-Ste50 complex is assembled in yeast cells, consistent with previous observations (Figure 2C) [12], [13]. In ste5Δ cells, the Ste11–Ste50 subcomplex was assembled normally. Similarly in ste50Δ mutants we efficiently recruited the pair Ste5–Ste11. Therefore, neither Ste5 nor Ste50 are required to maintain the assembly of the rest of the complex. However, in strains harboring a *STE11* knockout Ste5 and Ste50 were not co-recruited, suggesting that this interaction requires the presence of Ste11. Our observations are consistent with a central role of Ste11, which would establish interactions with the rest of the complex, and a more peripheral localization of Ste5 and Ste50, each of them interacting only with Ste11 [12], [14].

### Automated screening for PPIs

**Figure 3:**
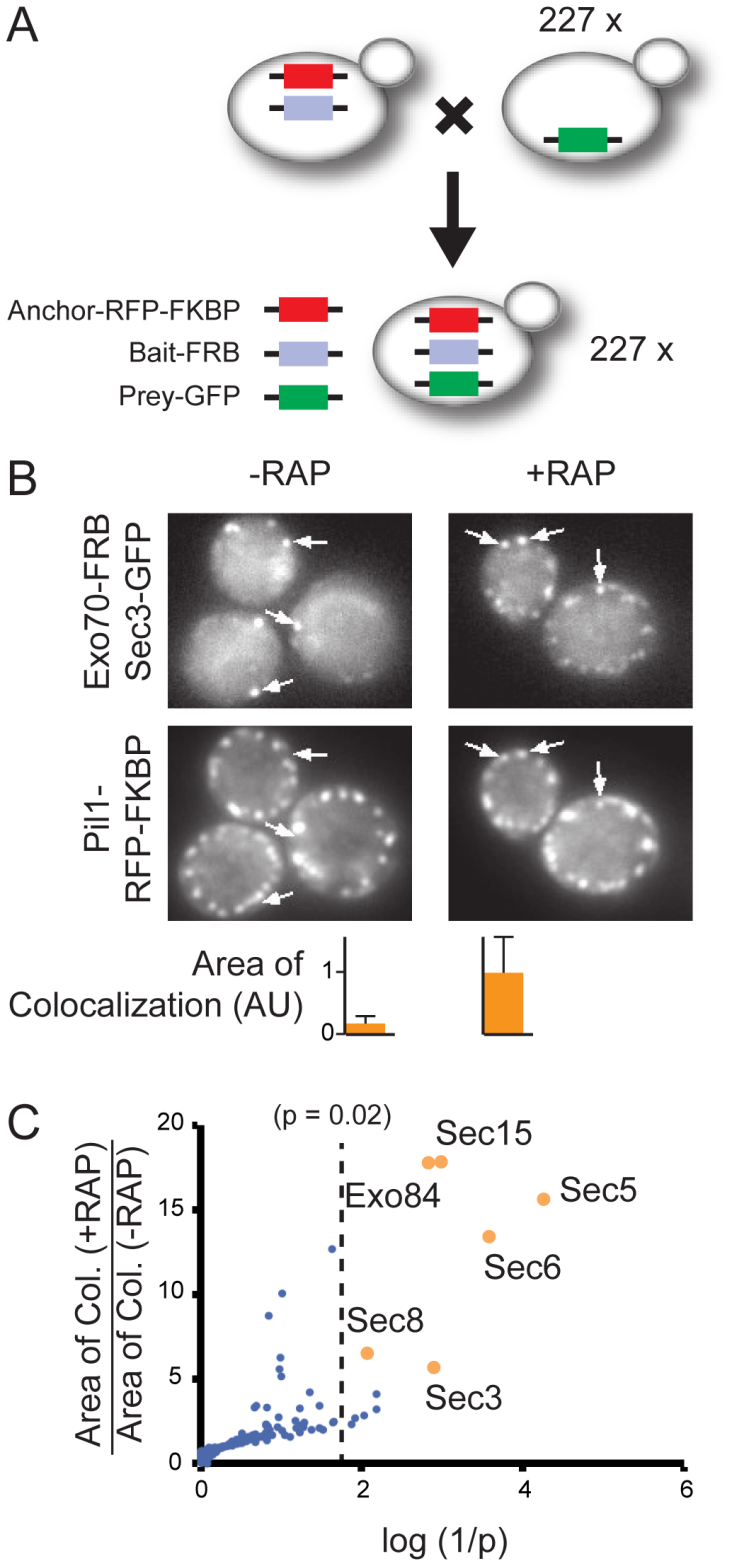
Automated PICT assay. (A) Schematic representation of strain generation. (B) Representative cells of the automated PICT assay from the screen for PPIs of Exo70. Cells were treated with the vehicle (−RAP) or rapamycin (+RAP). The upper images show the GFP channel and the bottom images show the RFP channel. Arrows highlight membrane sites where Sec3-GFP accumulates. The bars quantify the area of colocalization between Sec3-GFP and Pil1-RFP-FKBP from nine different fields of views (see Materials and Methods). (C) Summary of the screen. The Y-axis shows the ratio between the area of colocalization after and before Exo70-FRB recruitment. The X-axis shows the log (1/p) of the t-test between the area of colocalization before and after rapamycin treatment. All known exocyst components (orange circles) showed significant recruitment (log1/p>1.699; dashed line); other tested preys are represented as blue circles.

Importantly for large-scale studies, PICT can be easily automatized for high-throughput experiments. We used PICT to screen for PPIs of Exo70, an essential subunit of the exocyst, which is an octameric complex involved in exocytosis [15]. We used synthetic genetic array (SGA) technology to mate a yeast strain harboring the anchor (Pil1-RFP-FKBP) and bait (Exo70-FRB) with 227 prey strains from the GFP-tagged strain collection [16] (Figure 3A and Dataset S1). The preys were selected based on functional annotation as protein kinases, protein phosphatases, signal transducers, motor proteins or GTPases. In addition, six of the known subunits of the exocyst (Sec3, Sec5, Sec6, Sec8, Sec15 and Exo84) were included. The resulting 227 strains were grown in 96 well plates and images were taken automatically in the presence or the absence of rapamycin (Materials and Methods). Unlike the Ste5–Ste11–Ste50 complex, the exocyst normally localizes to both the cytosol and to patches on the cell surface. Therefore, to distinguish between the normal and rapamycin-induced localizations, we quantified the co-recruitment efficiency of GFP-tagged preys by measuring the area of colocalization between Pil1-RFP-FKBP and the GFP-tagged proteins for both treatments (Figures 3B and C and Materials and Methods). The screen identified six proteins interacting with Exo70, all the exocyst subunits present in our collection, demonstrating the potential of the approach (Figure 3C).

### Quantification of complex disassembling *in vivo*

**Figure 4:**
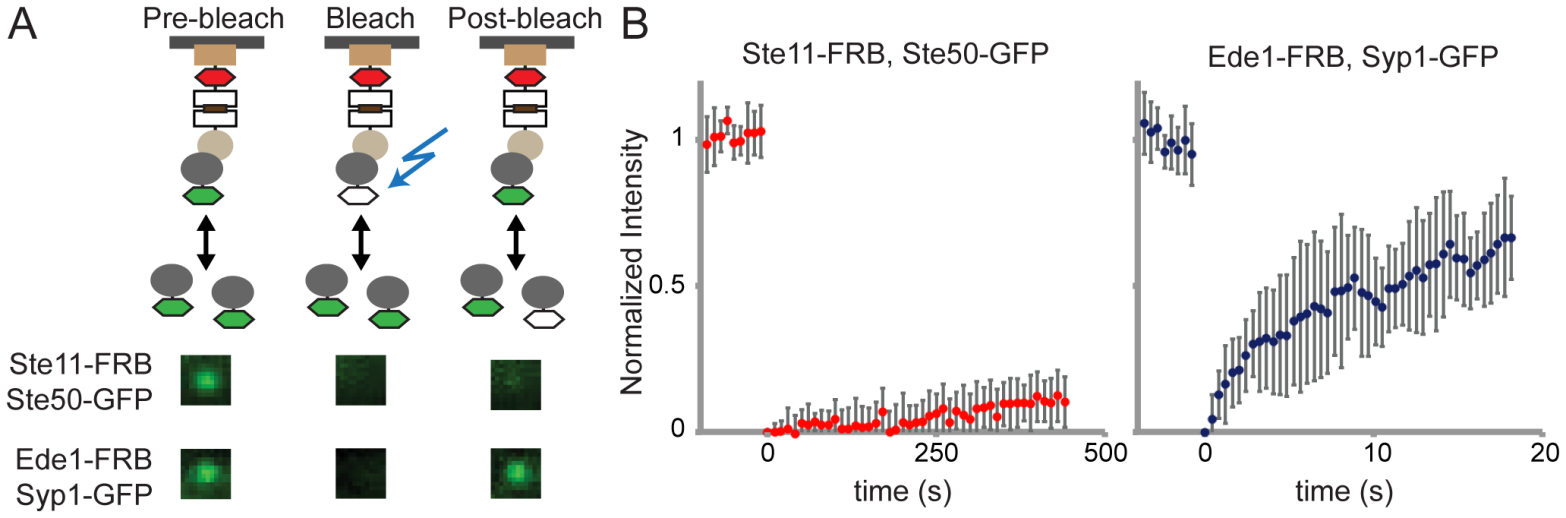
PICT-FRAP assay. (A) PICT-FRAP assay to analyze stable and transient interactions. A schematic representation of PICT-FRAP assay color-coded as in Figure 1. Ste11-FRB and Ede1-FRB were used as bait and Ste50-GFP and Syp1-GFP as prey in the respective experiments. For each assay, a frame from the GFP channel is shown corresponding to an anchoring site before, immediately after photobleaching and at the end of the measurements. (B) PICT-FRAP of the Ste11–Ste50 interaction (left) and the Ede1–Syp1 interaction (right). The curves represent the mean ± SD, Ste11–Ste50 (n = 8) and Ede1–Syp1 (n = 12).

Interestingly, since PICT is a live cell imaging method it can also be combined with other advanced light microscopy techniques to further characterize protein complexes in living cells. To demonstrate this we integrated FRAP with PICT (PICT-FRAP) to allow quantitative analysis of transient PPIs in vivo. Importantly, in PICT the bait proteins are stably recruited to Pil1 anchoring platforms and show no measurable exchange during our experiments (Video S2 and data not shown). However, prey proteins are co-translocated to the anchor site via their normal interactions and are therefore likely to preserve their binding dynamics. FRAP can be used to quantify the exchange of GFP-tagged preys recruited by a specific bait protein as a measure of the PPI dissociation rate. Thus, PICT-FRAP provides a general approach to study protein complex dissociation rates. We evaluated PICT-FRAP by analyzing two complexes: The interaction between Ste11 and Ste50, and the interaction between the endocytic proteins Ede1 and Syp1 [17]. Using PICT-FRAP we demonstrated that Ste11-FRB and Ste50-GFP form a stable complex with minor exchange during 7.5 minutes (Figures 4A and B, Video S3). Next, we translocated Ede1-FRB and Syp1-GFP to anchoring platforms (Figure S1). Syp1-GFP showed a fast exchange denoting short-lived interaction between the two proteins in agreement with what was detected previously at endocytic sites (Figures 4A and B, Video S4 and [18]).

## Conclusions

PICT is a simple approach available for any laboratory with a fluorescence microscope. Since it is not required to isolate the molecular assembly prior to analysis, PICT is an easy alternative method for the study of complexes that are difficult to purify. Additionally, given that the observations are fast and performed directly in living cells, it provides access to dynamic processes under normal and perturbed cellular conditions. Importantly, PICT can be easily applied in large-scale studies and it can be combined with other microscopy approaches such as FRAP to obtain quantitative measurements of protein complexes *in vivo*.

## Materials and Methods

### Plasmids

Plasmids generated in this study are listed in Table S1. All constructs were generated using conventional PCR, and molecular cloning methods. Briefly, the plasmids encoding the C-terminal tagging cassettes were designed to contain the S2/S3 primer annealing sites for PCR-targeting [19]. To generate pMK0067, the FRB (T2098L) sequence was amplified from the pLck-FRB*-eCFP plasmid (Carsten Schultz group, EMBL Heidelberg) with the primers TAGTATGTCGACGGTGGCGGTTCTGGTGGAATCCTCTGGCATGAGATGTG (Fw) and TAGTATGGATCCTCACTTTGAGATTCGTCGGAACACATG (Rv) and cloned into the SalI/BamHI site of pFA6a-hphNT1 vector [19]. To construct pMK0069, the FRB (T2098L) sequence was amplified from the pLck-FRB*-eCFP plasmid (Carsten Schultz group, EMBL Heidelberg) with the primers TAGTATGTCGACGGTGGCGGTTCTGGTGGAATCCTCTGGCATGAGATGTG (Fw) and TAGTATGGATCCCTTTGAGATTCGTCGGAACACATG (Rv) and cloned into the SalI/BamHI site of pYM12 monomeric GFP [20].

Finally, the FKBP sequence was amplified from the pGgamma2-FKBP-mRFP plasmid (Carsten Schultz group, EMBL Heidelberg) with the primers TAGTATGTCGACGGTGGCGGATCTGGAGGTGGATCAGGTGGAGGTTCAGGTGGTGGATCTGGTGGAATGGGAGTGCAGGTGGAAAC (Fw) and TAGTATGGATCCTTCCAGTTTTAGAAGCTCCACATC (Rv), cloned into the SalI/BamHI site of the pFA6a-natNT2 vector [19] and subsequently the mCherry-coding sequence was cloned in the SalI site to generate the pMK0080 plasmid.

### Yeast Strains

To obtain rapamycin resistant strains we replaced the endogenous *FPR1* gene with a kanMX4 cassette and incorporated the *tor1*-*1* point mutation into the *TOR1* gene by homologous recombination using the following primers:

Fw: GTTAGTCACGAGTTGATCAGAGTAGCCGTTCTATGGCACGAATTATGGTATGAAGGACTGGAAGATGCG**AGa**CGCCAATTTTTCGTTGAACATAACATAGAAAAAATGTTTTCTACTTTAGAACCTTTACATAAACACTT.

Rv: AAGTGTTTATGTAAAGGTTCTAAAGTAGAAAACATTTTTTCTATGTTATGTTCAACGAAAAATTGGCG**tCT**CGCATCTTCCAGTCCTTCATACCATAATTCGTGCCATAGAACGGCTACTCTGATCAACTCGTGACTAAC.

Positive colonies were selected on YPD plates containing 100 nM rapamycin (Sigma) and confirmed by sequencing.

S. cerevisiae strains used in this study are summarized in Table S2 and Dataset S1. Yeast genes were tagged or deleted at their genomic loci by PCR-based gene targeting [19]. The correct chromosomal integration was checked by PCR.

To screen for components of the exocyst complex the strain MKY2132 was crossed with 227 strains of the C-terminally GFP-tagged genomic collection [16] using SGA technology [21] (Dataset S1).

### PICT

All PICT assays were performed with the anchor Pil1-RFP-FKBP in yeast strains harboring the *tor1*-*1* mutant and lacking the endogenous FPR1 gene (see above). Analyzed strains were grown in synthetic defined (SD) medium with appropriate supplements at 25°C O.N. and diluted and grown next morning up to exponential phase. Cells attached on 35 mm glass bottom culture dishes coated with Concanavalin A were treated either with vehicle (DMSO) or 10 µM rapamycin (Sigma). Imaging was performed, after 15 min incubation, with an Olympus IX81 microscope equipped with 100×/NA 1.45 objective lens and Hamamatsu Orca-ER camera. Automated PICT was performed with the anchor Pil1-RFP-FKBP and the bait Exo70-FRB in 227 strains that contained a variety of different C-terminally GFP-tagged preys as candidates to participate in the exocyst complex. Six of the known components of the complex were also included: Sec3, Sec5, Sec6, Sec8, Sec15 and Exo84. The 227 strains were grown in SD media at 25°C O.N. in 96 well plates containing several controls: a strain expressing Sec5-GFP as a positive control for the PICT assay; a strain expressing Slm1-GFP (*yil105c*-GFP), a protein that normally colocalizes with the anchor Pil1 [22], as a positive control for the colocalization analysis; and a strain expressing Sla1-GFP (*ybl007c*-GFP), an endocytic protein present in different membrane structures than Pil1, and that does not participate in the exocyst complex [23], as a negative control for the colocalization analysis. Cells were attached on glass bottom 96 well plates coated with Concanavalin A. Prior to imaging, cells were treated either with vehicle (DMSO) or 10 µM rapamycin (Sigma) for 15–60 min. Automated imaging was performed with an automated Olympus IX81 microscope using an in-house modified version of Olympus Biosystems ScanR software and an image-based autofocus routine. For each sample nine fields of view were acquired.

### FRAP

The FRAP experiments were done with a custom-built set-up that focuses the 488 nm laser beam at the sample plane. The CCD camera, the filter wheels and the shutters were controlled by Metamorph software (Universal Imaging).

### Image analysis

ImageJ software (http://rsb.info.nih.gov/ij/) was used for general manipulation of images and movies. Image analysis was carried out using custom-written ImageJ macros. Images were background subtracted and all movies were corrected for photobleaching.

Manually acquired images were assessed visually for prey-GFP recruitment to anchoring sites. A prey was positively scored to interact with the bait-FRB when >90% of the membrane associated patches in the GFP channel colocalized with the RFP-FKBP-tagged anchor.

For the automated PICT screen the image analysis was performed with a custom-written ImageJ macro. Briefly, background was subtracted with the ImageJ “subtract background” command and a Rolling Ball Radius of 115 pixels. The red and the green channels were segmented using “auto local threshold” function with the following parameters: Radius = 5, Parameter 1 = −12, Parameter 2 = 0. The threshold was used in each channel to generate a mask that specifically selected small fluorescent patches such as the anchoring sites. The area of colocalization between the anchoring platforms (Pil1-RFP-FKBP, red channel) and the preys (GFP-tagged, green channel) was measured as the number of pixels included in the intersection of the two masks. Rapamycin treatment under our conditions does not affect the localization or the expression levels of Pil1 (results not shown). Therefore, we used the area included in the red channel mask, which is proportional to the number of cells, to normalize the area of intersection. To score the recruitment of each GFP-tagged prey, we measured the ratio between the area of colocalization upon treatment with rapamycin and the area of colocalization before treatment. A heteroscedastic t-test with one tail (n = 9 fields of view) was used to evaluate whether the increase in area of colocalization in the presence of rapamycin was significant (p<0.02 or log(1/p)>1.699). Although five more proteins (YAR019C, YLR096W, YLL010C, YKL079W and YOR008C) fulfilled this criteria, a visual inspection of the images indicated that their recruitment to anchoring sites was negligible.

## Supporting Information

Figure S1 **Recruitment of Ede1-Syp1 complex to Pil1-RFP-FKBP anchoring sites.** Ede1-FRB was used as a bait and Syp1-GFP as a prey. “–RAP” cells were treated with the vehicle, “+RAP” cells were treated with rapamycin. Arrows point to representative Syp1-GFP and Pil1-RFP-FKBP sites at the membrane. Co-localization only occurs upon rapamycin treatment. Images were taken on the surface of the yeast cells.
(TIF)

Table S1 **Plasmids used in this study.**
(DOC)

Table S2 **List of strains used in this study.**
(DOC)

Dataset S1 **Colocalization of prey-GFP and anchor-RFP-FKBP as a measurement of recruitment in automated screen for PPIs using PICT.**
(XLSX)

Video S1 **PICT of Ste5-Ste11-Ste50 complex of the pheromone-activated MAPK cascade.** Prey Ste50-GFP is co-recruited with bait Ste11-FRB upon addition of rapamycin at time 0s.
(MOV)

Video S2 **PICT-FRAP of the Pil1-RFP-FKBP and Exo70-FRB-GFP interaction induced by rapamycin.** Fluorescent recovery after photobleaching (FRAP) was applied to analyse the dynamics of the anchor-bait dimerization. No exchange was observed. The arrow points to the bleached anchoring platform.
(MOV)

Video S3 **PICT-FRAP of the Ste11-Ste50 interaction.** FRAP was applied to analyse the interaction dynamics between the prey Ste50-GFP and the bait Ste11-FRB. The arrow points to the bleached anchoring platform.
(MOV)

Video S4 **PICT-FRAP of the transient interaction between the endocytic proteins Ede1 and Syp1.** FRAP was applied to analyse the interaction dynamics between the prey Syp1-GFP and the bait Ede1-FRB. The arrow points to the bleached anchoring platform.
(MOV)
