# Supplementary figures and images for: Detection and Characterization of Protein Interactions In Vivo by a Simple Live-Cell Imaging Method
Source: PLoS One. 2013 May 1;8(5):e62195. doi: 10.1371/journal.pone.0062195 (PMC3641059; doi:10.1371/journal.pone.0062195)

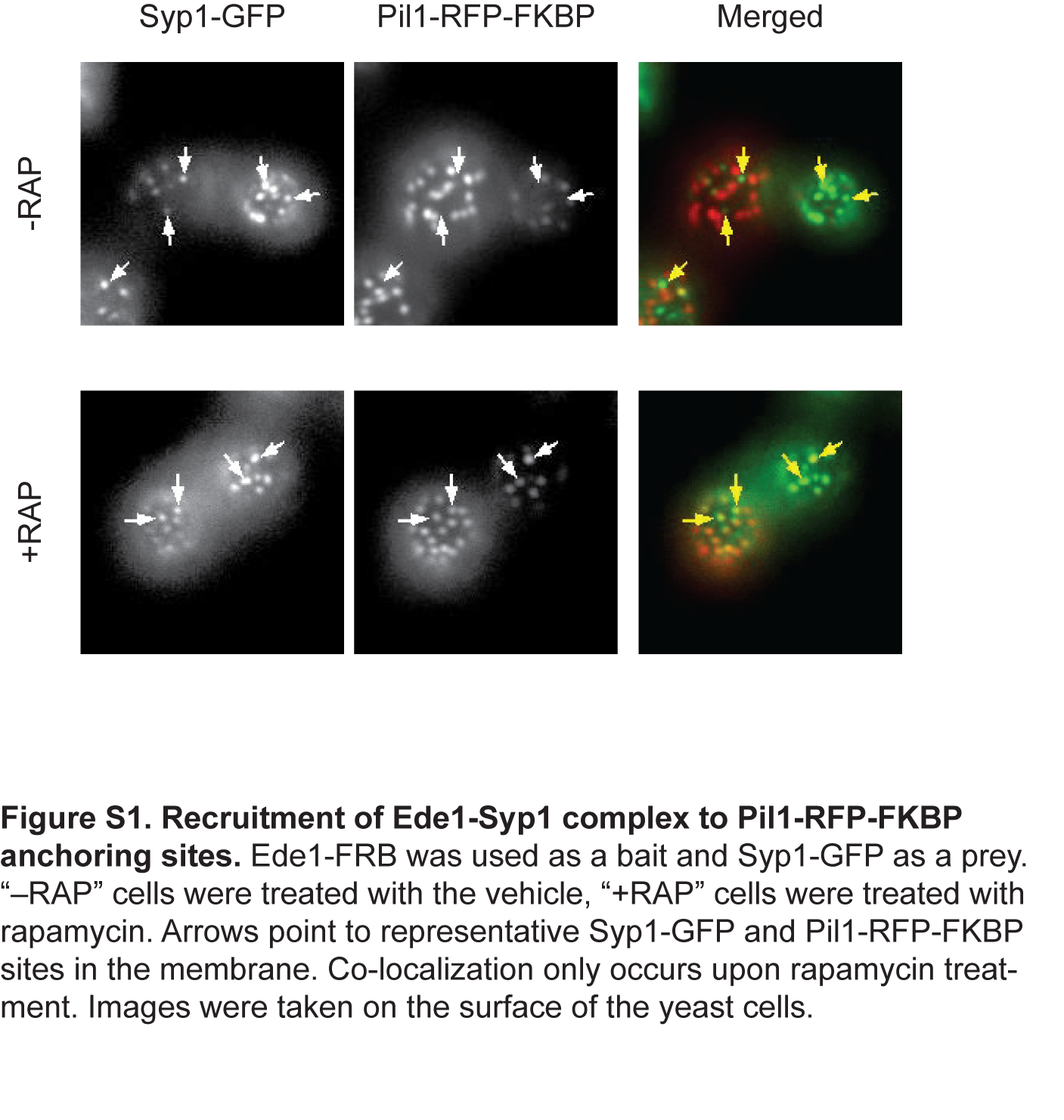

Supplement: Figure S1 — Recruitment of Ede1-Syp1 complex to Pil1-RFP-FKBP anchoring sites. Ede1-FRB was used as a bait and Syp1-GFP as a prey. “–RAP” cells were treated with the vehicle, “+RAP” cells were treated with rapamycin. Arrows point to representative Syp1-GFP and Pil1-RFP-FKBP sites at the membrane. Co-localization only occurs upon rapamycin treatment. Images were taken on the surface of the yeast cells. (TIF) [file pone.0062195.s001.tif]
