# Supplementary material for: Detection and Characterization of Protein Interactions In Vivo by a Simple Live-Cell Imaging Method
Source: PLoS One. 2013 May 1;8(5):e62195. doi: 10.1371/journal.pone.0062195 (PMC3641059; doi:10.1371/journal.pone.0062195)
Supplement: Table S1 — Plasmids used in this study. (DOC) [file pone.0062195.s002.doc]

**Table S1: Plasmids used in this study.**

| Plasmid | Tag | Linker (amino acids) | Marker | Source |
| --- | --- | --- | --- | --- |
| pMK0067 | FRB(T2098L) | 12 | Hph | This study |
| pMK0069 | FRB(T2089L)-meGFP | 12 | Kan | This study |
| pMK0080 | RFP-FKBP | 24 | Nat | This study |
