# Supplementary material for: Detection and Characterization of Protein Interactions In Vivo by a Simple Live-Cell Imaging Method
Source: PLoS One. 2013 May 1;8(5):e62195. doi: 10.1371/journal.pone.0062195 (PMC3641059; doi:10.1371/journal.pone.0062195)
Supplement: Table S2 — List of strains used in this study. (DOC) [file pone.0062195.s003.doc]

**Table S2: List of strains used in this study.**

| **Strain** | **Genotype** | **Source** |
| --- | --- | --- |
| BY4741 | MATa, *his3Δ1, leu2Δ0, met15Δ0, ura3Δ0* | Invitrogen |
| MKY0100 | MATa, *his3200, leu2-3,112, ura3-52, lys2-801* | David Drubin |
| SGAY7039 | MATα, *his3Δ1 leu2Δ0 ura3Δ0 LYS+, can1Δ::Ste2pr-Leu2, lyp1Δ::* | Charles Boone |
| MKY2127 | SGAY7039 *tor1-1* | This study |
| MKY2128 | MKY2127 *fpr1Δ::klURA* | This study |
| MKY2131 | MKY2128 *PIL1-RFP-FKBP::natNT2* | This study |
| MKY2132 | MKY2131 *EXO70-FRB::hphNT1* | This study |
| MKY1490 | MKY0100 *tor1-1* | This study |
| MKY1585 | MKY1490  *fpr1Δ::klURA* | This study |
| MKY2181 | MKY1585  *PIL1-RFP-FKBP::natNT2* | This study |
| MKY2421 | MKY2181 *STE5-FRB::hphNT1* | This study |
| MKY2423 | MKY2181 *STE11-FRB::hphNT1* | This study |
| MKY2668 | MKY2181 *STE50-FRB::hphNT1* | This study |
| MKY2751 | MKY2421 *STE11-3xmyeGFP::kanMX4* | This study |
| MKY2752 | MKY2421 *STE50-3xmyeGFP::kanMX4* | This study |
| MKY2431 | MKY2423 *STE5-3xmyeGFP::kanMX4* | This study |
| MKY2432 | MKY2423 *STE50-3xmyeGFP::kanMX4* | This study |
| MKY2754 | MKY2668 *STE5-3xmyeGFP::kanMX4* | This study |
| MKY2755 | MKY2668 *STE11-3xmyeGFP::kanMX4* | This study |
| MKY2756 | MKY2751 *fpr1Δ::* | This study |
| MKY2757 | MKY2752 *fpr1Δ::* | This study |
| MKY2758 | MKY2431 *fpr1Δ::* | This study |
| MKY2759 | MKY2432 *fpr1Δ::* | This study |
| MKY2760 | MKY2754 *fpr1Δ::* | This study |
| MKY2761 | MKY2755 *fpr1Δ::* | This study |
| MKY2762 | MKY2756 *ste50Δ::klURA* | This study |
| MKY2763 | MKY2757 *ste11Δ::klURA* | This study |
| MKY2783 | MKY2758 *ste50Δ::klURA* | This study |
| MKY2784 | MKY2759 *ste5Δ::klURA* | This study |
| MKY2785 | MKY2761 *ste5Δ::klURA* | This study |
| MKY2786 | MKY2760 *ste11Δ::klURA* | This study |
| MKY2769 | MKY2181 *STE11-FRB-myeGFP::kanMX4* | This study |
| MKY2777 | BY4741  *PIL1-RFP-FKBP::natNT2*, *EXO70-FRB-myeGFP::kanMX4* | This study |
| MKY2595 | MKY2181 *EDE1-FRB::hphNT1,*  *SYP1-yeGFP::HIS3MX6* | This study |
